# Content-independent embedding scheme for multi-modal medical image watermarking

**DOI:** 10.1186/1475-925X-14-7

**Published:** 2015-02-04

**Authors:** Hussain Nyeem, Wageeh Boles, Colin Boyd

**Affiliations:** Department of Electronics and Communication Engineering, Khulna University of Engineering and Technology (KUET), 9203 Khulna, Bangladesh; School of Electrical Eng. & Computer Science, Queensland University of Technology (QUT), Brisbane, 4001 Queensland Australia; Department of Telematics, Norwegian University of Science and Technology (NTNU), 7491 Trondheim, Norway

**Keywords:** Multi-modality medical images, General RONI selection, Content-independent embedding, Fragile watermarking

## Abstract

**Background:**

As the increasing adoption of information technology continues to offer better distant medical services, the distribution of, and remote access to digital medical images over public networks continues to grow significantly. Such use of medical images raises serious concerns for their continuous security protection, which digital watermarking has shown great potential to address.

**Methods:**

We present a content-independent embedding scheme for medical image watermarking. We observe that the perceptual content of medical images varies widely with their modalities. Recent medical image watermarking schemes are image-content dependent and thus they may suffer from inconsistent embedding capacity and visual artefacts. To attain the image content-independent embedding property, we generalise RONI (region of non-interest, to the medical professionals) selection process and use it for embedding by utilising RONI’s least significant bit-planes. The proposed scheme thus avoids the need for RONI segmentation that incurs capacity and computational overheads.

**Results:**

Our experimental results demonstrate that the proposed embedding scheme performs consistently over a dataset of 370 medical images including their 7 different modalities. Experimental results also verify how the state-of-the-art reversible schemes can have an inconsistent performance for different modalities of medical images. Our scheme has MSSIM (Mean Structural SIMilarity) larger than 0.999 with a deterministically adaptable embedding capacity.

**Conclusions:**

Our proposed image-content independent embedding scheme is modality-wise consistent, and maintains a good image quality of RONI while keeping all other pixels in the image untouched. Thus, with an appropriate watermarking framework (*i.e.*, with the considerations of watermark generation, embedding and detection functions), our proposed scheme can be viable for the multi-modality medical image applications and distant medical services such as teleradiology and eHealth.

## Introduction

Advances in adoption of modern information technology has enabled the healthcare organisations to offer various distant medical services (*e.g.*, teleradiology, eHealth). Those services allow remote access to, and electronic transmission and interpretation of, medical images across multiple users and display stations. While such uses of medical images offer distinct opportunities of improving healthcare access, delivery, and standards, security protection of the images throughout their lifetime becomes more challenging. Many new security problems and legal and ethical concerns (*e.g.*, image fraud, distrust and invasion of privacy) are emerging, which digital watermarking has shown a great potential to address [1, 2].

Digital watermarking is an evolving data-hiding technology that has three main components: watermark- generation, -embedding and -detection [3, 4]. Watermark generation generates watermarks from its input information including medical images and other radiological information (*e.g.*, EPR—electronic patient records). Watermark embedding embeds the watermarks in medical images such that they can be detected later by the watermark detection. Thereby, the authenticity and integrity of the images can be verified and the meta-data (*e.g.*, EPR) can be imperceptibly annotated in the images [1]. Although this may also require the watermark generation to employ a suitable cryptographic technique (*e.g.*, encryption, digital signature), in this paper, we restrict our focus on the watermark embedding process.

Watermark embedding has to meet a few strict requirements for medical images [5], since it incurs an inevitable distortion. They are: (*i*) continuous protection of the image, and (*ii*) “acceptable” embedding distortion in the image. Continuous protection of a medical image requires the watermark to be always embedded in the image. Additionally, an acceptable embedding distortion guarantees the reliability of a watermarked image for any medical or clinical uses. To meet these requirements, two types of embedding schemes are mainly studied: (*i*) lossless compression of ROIs (regions of interest, to medical professionals) [6, 7] and (*ii*) reversible embedding [8–16].

The reversible embedding schemes seem to be relatively advantageous, since they usually can avoid the ROI segmentation (considering the original image can be restored) and compression. Reversible embedding introduces an invertible distortion in a watermarked medical image that can be restored to the original, when required (*e.g.*, for medical diagnosis purpose). It has different principles and properties that underpin many reversible watermarking schemes for medical image applications [17]. We briefly review some prominent reversible schemes in Section “State of medical image watermarking”.

However, the general problem of the reversible schemes is that their watermark embedding process is image- content dependent (*i.e.,* depends on the perceptual or visual content of the input images). Thereby, they seem to suffer from an inconsistent performance for different modality^a^ medical images. Particularly, for a given embedding capacity requirement, the embedding time and distortion may widely vary with the image modality. Consequently, a reversible scheme while may perform well for a particular modality, it may not be equally suitable for other modalities. (We note that the image-content dependent property may be required by the watermark generation, which is beyond the scope of this paper and should not be confused with the watermark embedding.)

Therefore, as a primary contribution of this paper, we present a content-independent embedding scheme for medical images (Section “Proposed embedding scheme”). We generalise the RONI (region of non-interest; complementary region of ROI) selection that offers content-independent embedding and avoids the computational overhead of ROI segmentation. Our proposed scheme further attempts to find a set of suitable least significant bit-planes (LSB-planes) to maintain a good (perceptual) quality in the embedding region (*i.e.*, RONI), keeping the other pixels in the image (*i.e.*, in ROI) untouched. We present our experimental results to verify how the reversible embedding affects the watermarked image quality, and thereby validate our proposed scheme, for different medical image modalities (Section “Experimental results and discussion”).

## State of medical image watermarking

Reversible watermarking is arguably most suitable for the medical image applications. Many reversible watermarking schemes have been reported in the literature, since the Barton patent [8] in 1997. However, the difference expansion (DE) [10] and histogram shifting (HS) [14] based reversible watermarking and their recent developments [11, 12, 15, 16, 18] have attracted increasing interest in medical image applications.

Coatrieux *et al.*[12] presented a reversible watermarking scheme to continuously protect the reliability of MR images. However, that scheme may not be suitable for the other modalities of medical images due to its morphology based RONI segmentation. Lee *et al.*[11] proposed a reversible watermarking scheme that employs adaptive embedding of watermark in the high frequency wavelet coefficients of the medical images for high embedding capacity with a low level distortion. Nayak *et al.*[18] proposed a technique of interleaving patient record in medical images using HS watermarking. Discontinuity (*i.e.*, when the watermarked image is restored to the original) of security protection and disregard of RONI embedding may reduce the practicability of those schemes [11, 18] for medical image applications.

Guo and Zhuang [15] presented a DE reversible watermarking scheme for medical images. That scheme considers a region based embedding to preserve the ROI; but, the manual ROI selection and information overhead of multiple polygons would possibly make the scheme less efficient. Tsai *et al.*[16] proposed a HS watermarking scheme for medical images by utilizing a linear prediction embedding that offers relatively high embedding capacity with a low-level distortion. Similar to the schemes in [11, 18], Tsai *et al.* scheme also does not consider the RONI for embedding, which may raise legal and ethical concerns about erratically altering medical images [5].

However, as the problem in question, most of the reversible schemes including the schemes in [11, 12, 15, 16, 18] have an image content-dependent embedding approach.

As mentioned in Section “Introduction”, for an image content-dependent embedding scheme, level of distortion in different modality medical images (even of the same sizes) may not be the same for embedding the same size watermark (or more precisely, the same size payload—the watermark plus any side information). This also means that the scheme may always require to check if the capacity is sufficient and the level of distortion is acceptable, for a given watermark.

Thus, the overall performance of such schemes would possibly vary with the modality of medical images. Moreover, for reversible schemes, once the watermarked image is restored to the original, any security protection discontinues.

In addressing the above limitations in the state-of-the-art medical image watermarking schemes, in this paper, we propose a content-independent embedding scheme that utilises the LSB-planes of the RONI.

We substantially extend our earlier work [5, 19] to demonstrate the potential of the content-independent embedding, and its applicability to multi-modality medical images.

We generalise the RONI selection and determine its criteria to facilitate the content-independent embedding. Further, we investigate the influence of the content-dependent embedding approach of a prominent medical image watermarking scheme [16] for different modalities of medical images. Thereby, we verify the consistency in performance of our proposed scheme and validate its applicability to multi-modality medical images.

## Proposed embedding scheme

We now present the development of the content-independent embedding scheme. We first generalise the RONI selection for different modalities of medical images. Utilising LSB-planes of the RONI will then help us to achieve the image content-independent capacity and continuous security protection (using standard cryptographic techniques, as mentioned in Section “Introduction”).

### Generalising RONI selection

Generalising RONI selection is a challenging task. Ideally, ROIs in medical images are both modality- and patient-wise uncorrelated, which make them more or less a random phenomenon. For example, two head-MRIs of two different patients can have different ROIs. A subjective RONI selection [6, 7, 20–22] is thus often used (*e.g.*, manual ROI selection by a doctor), which reduces the embedding performance (*e.g.*, increase the computation time).

Other RONI selection techniques [12, 15, 23–27] (*e.g.*, using morphological operation, logical rectangle, ellipse, polygon, *etc.*) can tackle this problem, but only to certain modalities.

A general RONI selection technique is still lacking in the literature.

In order to conceptualise the general RONI selection process, we thus start with looking at the individual modality medical images and their usual ROIs’ locations. Necessary considerations are made based on the observations, then RONI selection criteria are specified, and the capacity control parameters are determined.

Thereby, a general RONI selection approach is developed below.

#### Observations on the general ROI location

We propose the border pixels of medical images as the general RONI considering ROI location to be clustered around the centre of the images.

**Figure 1:**
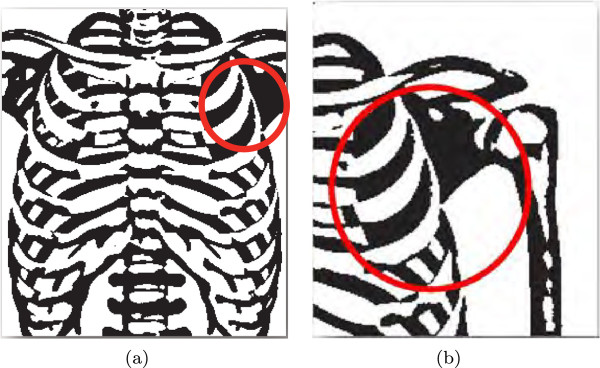
**Examples of ROI location in the medical image.** **(a)** close to the border, and **(b)** close to the center [19].

Irrespective of the modalities and parts of human body under examination, a general consideration in acquiring medical images is to endeavour to keep the prospective ROIs at the centre of the images [28]. For example, consider the scenario presented in Figure 1. Suppose the ROI of a chest X-ray shown in Figure 1(a) was found close to the border of the image as indicated by the red-circle, where the doctor (and/or medical specialist) being prudent usually requests another image that places the ROI closer to the centre and away from the border as shown in Figure 1(b). This fact suggests that the border-pixels of medical images are of little or no significance to the doctors and can be generally used as RONI. The border-pixels of a medical image thus can be conceptualised as the general RONI based on the following facts [19]:

- For a medical image, the phenomenon under examination by the doctors or other medical practitioners would occupy the central part of the image.
- In situations, where ROI gets close to the border of the image, the examiner is expected to seek another image, where the ROI would be placed away from the border and closer to the centre of the image.

#### The RONI selection criteria

A set of RONI selection criteria is now to be specified for proceeding with the considerations mentioned above. These criteria would help determine the suitability of the RONI selection approach for the medical images. As we determined in [19], based on the medical image watermarking requirements, the RONI selection should meet the following criteria:

- RONI should have no relevance or impact on the medical objectives for which the image was obtained.
- RONI should provide the required capacity to accommodate the payload.
- RONI should keep the distortion at the “minimum” level.
- RONI should have the “minimum” computational and side-information overhead.

Our scheme can be expected to perform reasonably well for each criterion. The selected RONI (*i.e.,* border pixels) in our scheme can have minimum impact on the medical objectives, since those pixels of a medical image are considered to be less significant for the medical uses (for example, see [28]). Our scheme is also expected to provide better flexibility (with a set of capacity control parameters, as discussed in the following section) for increasing payload size and consistency in performance (in terms of PSNR, MSSIM, and embedding time). Additionally, since our RONI selection avoids any segmentation and compression, it should generally be faster than other RONI selection processes for different modality medical images.

#### Capacity control parameters

Determining the capacity parameters for the capacity control is also required for using the border- pixels in RONI selection. Here, capacity control is the process used to attain the required embedding capacity with the lowest level of possible distortion. In order to have an efficient capacity control, we thus consider two parameters, namely; *border-width*, *N*_*BW*_ and *bit-depth*, *N*_*LSB*_. The border-width specifies how many pixels in the border of an image can be suitably used as RONI, and the bit-depth specifies how many LSB-planes of the selected border-pixels can be used for embedding the payload, while keeping distortion at the minimum level. Depending on the required capacity, the values of these parameters are determined using the capacity control of the embedding scheme.

Thus our embedding scheme can adaptively satisfy the capacity requirement.

### Development of our proposed embedding scheme

**Figure 2:**
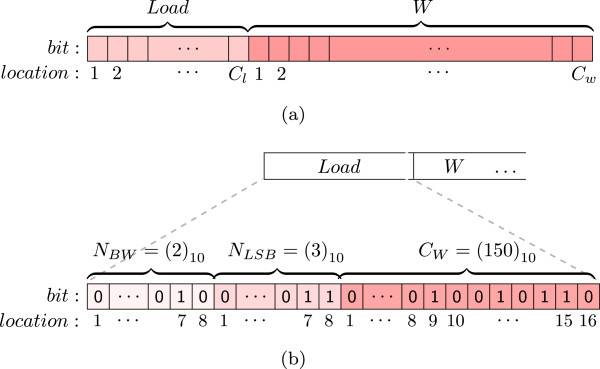
**Data-frame : (a) payload,** ***P*** **, and (b)** ***Load*** **for** ***C***_***l***_ ***= 32*** **.**[5, 19].

We now present our proposed embedding scheme based on the above generalised RONI selection principle. The scheme adaptively uses the LSBs of the RONI, and thus facilitates the image content-independent embedding. Specifically, for a given medical image, *I* and a watermark, *W*, the scheme determines the optimum combination of *N*_*BW*_ and *N*_*LSB*_ for embedding the payload, *P*. This ensures that the embedding distortion in RONI remains at a minimum level, satisfying the *capacity condition*[19] in equation 3. Here *C*_*total*_ is the total capacity and *C*_*p*_ is the size of payload, where *C*_*total*_ and *C*_*p*_ are are calculated by using 2 and 1 as shown in [19]; and, *r* and *c* are the numbers of pixels in a row and in a column of an input image, respectively. The function *Size*(·) determines the bit-length of its input. As shown in Figure 2(a), the *Load* is the formatted side information.
1

23

In order to find the optimum combination of *N*_*BW*_ and *N*_*LSB*_, until the capacity condition is satisfied, *N*_*BW*_ and *N*_*LSB*_ (initialised at value one, ‘1’) are increased by a unit step to increase the *C*_*total*_. We observed (from our experiments presented in our previous work [5, 19]) that increasing *N*_*BW*_ gives higher capacity for a fixed *N*_*LSB*_ than increasing *N*_*LSB*_ for a fixed *N*_*BW*_. Therefore, firstly, *N*_*BW*_ is increased successively (after checking the capacity condition each time) up to its given maximum usable limit, *T*_1_. Then, *N*_*LSB*_ is increased by a unit step, when *N*_*BW*_=*T*_1_. In this way, until the condition in 3 is fulfilled, *N*_*BW*_ and *N*_*LSB*_ are increased up to their maximum usable limits *T*_1_ and *T*_2_, respectively.

The threshold pair (*T*_1_,*T*_2_) has an important role to select the RONI in terms of *N*_*BW*_ and *N*_*LSB*_, and thus to adaptively control the capacity for an increasing size of watermark. A well defined (*T*_1_,*T*_2_) is required for all modality medical images to accommodate the usual size of watermarks used in a particular application. However, for a given (*T*_1_,*T*_2_), if the capacity condition is not satisfied (*i.e.*, no *N*_*BW*_ and *N*_*LSB*_ are found for the given watermark and image), a user prompt may be required to update (*T*_1_,*T*_2_), for any special cases.

Once the optimum *N*_*BW*_ and *N*_*LSB*_ are determined, the payload is embedded using the embedding function, *E*(·) as given in 4.

The function *E* (·) replaces the bits in the selected RONI’s LSB-planes. We summarise the steps of our embedding scheme below.

**Input:** (i) a medical image; and (ii) watermark

**Output** a watermarked image

**Step 1** We first compute the total capacity, *C*_*total*_ using 2.

**Step 2** Then we compute the payload size, *C*_*p*_, which is the size of the input watermark and predefined data-frame (in bits).

**Step 3** We find the optimum combination of *N*_*BW*_ and *N*_*LSB*_ to satisfy the capacity condition in 3. (An example of how to find the optimum values for *N*_*BW*_ and *N*_*LSB*_ is discussed in Section “An example of the proposed embedding scenario”.)

**Step 4** Once the optimum *N*_*BW*_ and *N*_*LSB*_ are determined for embedding, we compute the payload, *P* according the “data-frame” (will be discussed in Section “An example of the proposed embedding scenario”) shown in Figure 2.

**Step 5** We then embed the payload in the LSBs of RONI calculated by the computed minimum values of *N*_*BW*_ and *N*_*LSB*_, and thus obtain the watermarked image.

A detector requires *N*_*LSB*_, *N*_*BW*_, and the size of the watermark, *C*_*w*_, to extract the watermark independently. These information are formatted in a predefined data-frame of *Load* using a function, *Format*(·), as given in 5. Figure 2(b) illustrates an example of *Load* data-frame for a 32-bit of side information. First 8-bit is for *N*_*BW*_, next 8-bit is for *N*_*LSB*_, and the last 16-bit is for the watermark size, *C*_*w*_. It is worth noting that this structure may be re-defined according to the need for any watermarking objectives. Additionally, Figure 2(a) shows how *Load* is concatenated with the given watermark, *W* to compute the payload, *P*. A concatenation function, *Concat* (·) is given in 6 to concatenate its inputs in their given order and to output a single bit-stream.
456

As a part of detection, the embedded payload is extracted from an input watermarked image, . A detector initialises the predefined *Load* data-frame, and obtains the *N*_*BW*_, *N*_*LSB*_, and *C*_*w*_ from the extracted *Load* data. The detector then extracts the *C*_*w*_-bit from the *N*_*BW*_ border pixels of the *N*_*LSB*_ LSB-planes. Considering no bit-error occurs during the communication of , the extracted *C*_*w*_-bits should be the same as the embedded *W*-bits.

### An example of the proposed embedding scenario

**Figure 3:**
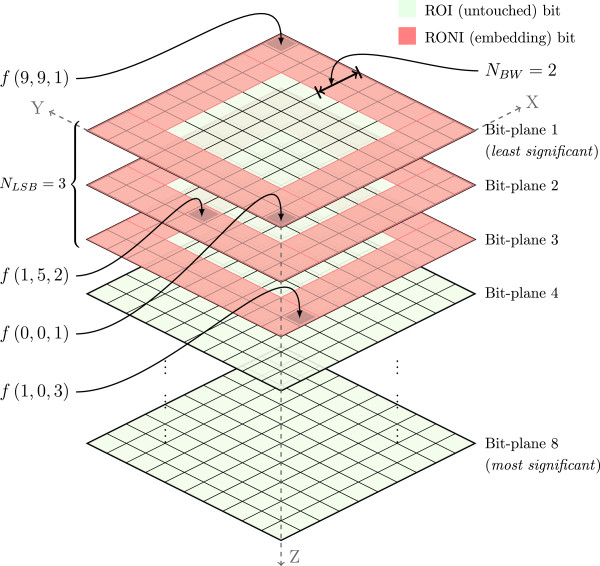
**An example of the proposed embedding scheme for an** ***8*** **-bit image of size 10** ***×*** **10 with** ***N***_***BW***_ ***= 2*** **and** ***N***_***LSB***_ ***= 3*** **.** (Few arbitrary bit locations in different bit planes, *e.g.*, *f*(0,0,1), *f*(1,5,2), *etc.* are shown to realise overall bit locations in the bit-planes.) [5, 19].

We illustrate here the computation of optimum *N*_*BW*_ and *N*_*LSB*_ and their use in our embedding scheme. Consider, an 8-bit image (of size 10×10) sliced into its 8-bit planes along *Z* - *axis*, as shown in Figure 3. Also consider, a 150-bit watermark (*i.e.*, *C*_*w*_= 150) is to be embedded and the given thresholds for *N*_*BW*_ and *N*_*LSB*_ are *T*_1_= 2 and *T*_2_= 4, respectively. With a 32-bit *Load* data frame (*i.e.*, *C*_*l*_= 32), *C*_*p*_ becomes 182-bit (*i.e.*, *C*_*p*_= 150 + 32 = 182). Now, to compute the optimum values for *N*_*BW*_ and *N*_*LSB*_ that meet the capacity condition in 3, individual steps of computing *C*_*t*_ using 2 for the given *T*_1_ and *T*_2_ are:

Once the capacity condition is met, the embedding function stops computation of *C*_*total*_. In this example, when *N*_*BW*_= 2 and *N*_*LSB*_= 3, total capacity, *C*_*total*_ becomes higher than the payload size, *C*_*p*_, and thus the capacity condition is satisfied. As Figure 2(b) shows, 32-bit *Load* is now computed for *N*_*BW*_= 2, *N*_*LSB*_= 3, *C*_*w*_= 150. (We note that a 32-bit *Load* data-frame in Figure 2(b) seems to be superfluous for a 125-bit watermark of this example. That frame in fact allows up to 2^16^= 65536-bit watermark, which can be redefined accordingly if required for any higher/lower size of watermark.)

Then, 182-bit *P* is computed by concatenating the *Load* and *W*, and finally embedded in the LSBs of RONI in a predefined embedding order. For example, embedding can be started from *f*(0,0,1) to *f*(5,8,3) that occupies 182-bits sequentially. The outer border pixels come first, then inside-border pixels successively and this follows up to *N*_*BW*_ pixels of all LSB-planes up to *N*_*LSB*_= 3.

In the detection process, the *Load* information is extracted from the given , and thus *N*_*BW*_= 2, *N*_*LSB*_= 3, and *C*_*w*_= 150 are obtained. As in the above example of embedding order, the watermark bits are extracted following the *Load* data-frame. In other words, from the very next bit location of the *Load* data-frame, 150 bits are extracted from 3 LSB-planes of 2 border pixels, which is the watermark, *W*.

## Experimental results and discussion

In this section, we verify and validate the consistent performance of the proposed watermark embedding for different modalities of medical image. We conducted several experiments to evaluate the performance of our scheme and compare it with that of the Tsai *et al.* scheme [16]. Choice of the Tsai *et al.* scheme is based on the prominence of the scheme which employs the state-of-the-art reversible embedding technique for different modality medical images. We used a set of 370 medical images of different modalities (*i.e.*, CT, MR, X-ray, DSA, US, RF and MG) and of different file formats (*e.g.*, DCM, DC3, JPG, BMP, *etc.*) Image sizes range from 196×258 to 600×600 (pixels), and image bit-depths are 8-bit and 16-bit. A watermark is considered as a set of binary arrays, {0,1}^+^. All necessary simulations were carried out in MATLAB (R2012a-7.140.739) and using an Intel Core i5 3.2 GHz CPU.

### Performance evaluation of the proposed scheme

**Figure 4:**
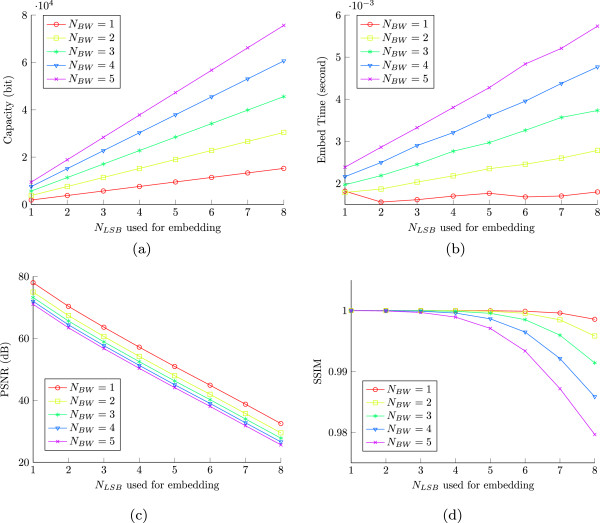
**Performance of the proposed embedding scheme: (a) capacity, (b) embedding time, (c) PSNR, and (d) MSSIM.**

We started our experiments with the performance evaluation of our scheme. Embedding capacity, watermarked image quality, and embedding time were considered as the performance evaluation parameters. We tested our scheme with *N*_*BW*_∈{1,2,⋯,5}, and *N*_*LSB*_∈{1,2,⋯,8}. Although *N*_*BW*_ and *N*_*LSB*_ can be increased in need of higher capacity requirement as discussed in Section “Development of our proposed embedding scheme”, we considered that the above limits of *N*_*BW*_ and *N*_*LSB*_ will be reasonable to verify and validate the consistent performance of the proposed scheme. As expected, the capacity, embedding time, and image quality degradation have increased gradually with the increase of *N*_*BW*_ and *N*_*LSB*_, as shown in Figure 4(a)–4(d).

Particularly, the adaptable embedding capacity of our scheme is verified. This means that the embedding capacity of our scheme can be controlled with changing *N*_*BW*_ and *N*_*LSB*_ as evident in Figure 4(a). Additionally, the embedding time varies almost linearly with the parameters, *N*_*BW*_ and *N*_*LSB*_. Figure 4(a) and 4(b) show that for higher *N*_*LSB*_, increasing *N*_*BW*_ has relatively high impact on the increasing capacity and embedding time. This suggests that increasing *N*_*BW*_ would be more effective to achieve a high capacity requirement.

On the other hand, the image quality of our scheme drops gradually with the increase of *N*_*BW*_ and *N*_*LSB*_. Figure 4(c) and Figure 4(d) show that the degradation in image quality (calculated over the whole image, although only border pixels are used for embedding) in terms of PSNR and MSSIM, respectively. The PSNR–*peak signal-to-noise ratio* estimates the perceived errors without any consideration of the subjective quality of an image. This means that the changes of the same number of LSBs in ROI and RONI (of a medical image) separately can have similar PSNR value, although they may have different perceptual outcomes. PSNR is therefore not suitable for our scheme, where almost all of the perceptually significant image pixels (in ROI) are left untouched in the proposed embedding.

In contrast to PSNR, a particularly designed image quality metric, MSSIM–*mean structural similarity* index illustrates a more reasonable relationship of image quality degradation with *N*_*BW*_ and *N*_*LSB*_, as shown in Figure 4(d). In fact, MSSIM measures the local similarity of perceptual contents thus it mainly accounts for the changes in perceptually significant information [29–31]. Therefore, we used MSSIM instead of PSNR for the performance comparison of our scheme, which will be discussed below in Section “Performance comparison”. The general formulation of MSSIM [29] is given below in 7.
78

where, *x*_*j*_ and *y*_*j*_ are the image content at *j*-th local window and their structural similarity index, *SSIM*(*x*_*j*_,*y*_*j*_) is computed using 8. Here, *μ*_*x*_ and *μ*_*y*_ are the average values of *x* and *y*, and  and  are the variance of *x* and *y*, respectively; *σ*_*xy*_ is the covariance of *x* and *y*; and *c*_1_= (*k*_1_*L*)^2^ and *c*_2_= (*k*_2_*L*)^2^ are two variables to stabilize the division with weak denominator for the *L* dynamic range of the pixel values. The default values of the weight factors, *k*_1_ and *k*_2_ are set to 0.01 and 0.03, respectively.

**Figure 5:**
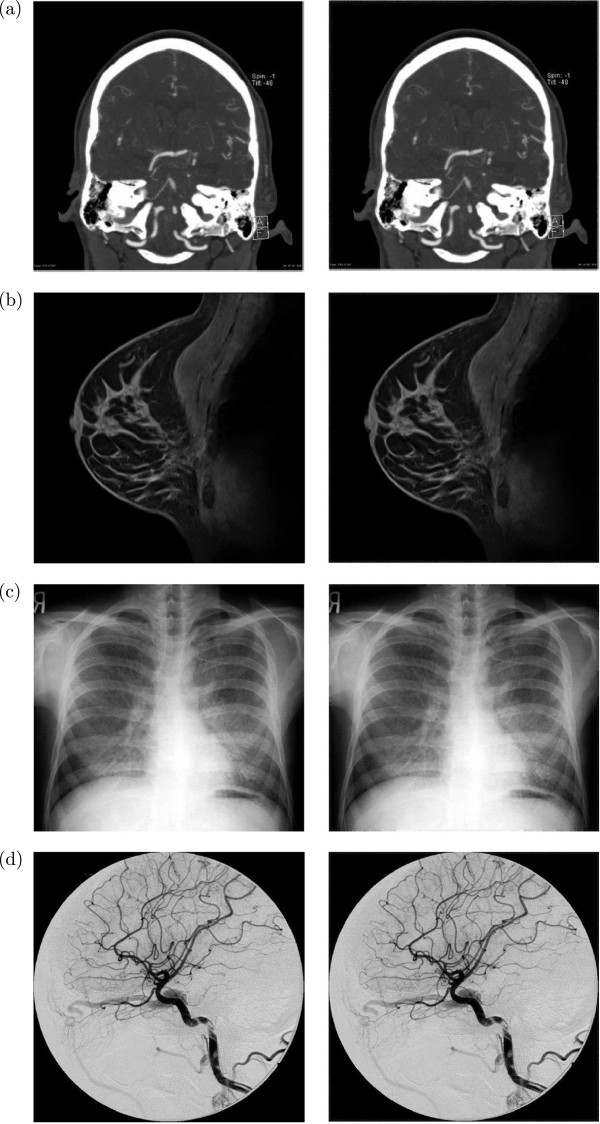
**Original (left column) and watermarked (right column, for** ***N***_***BW***_ ***=5*** **and** ***N***_***LSB***_ ***= 5*** **) versions of medical images of different modalities: (a) CT, (b) MR, (c) X-ray, and (d) DSA.**

**Figure 6:**
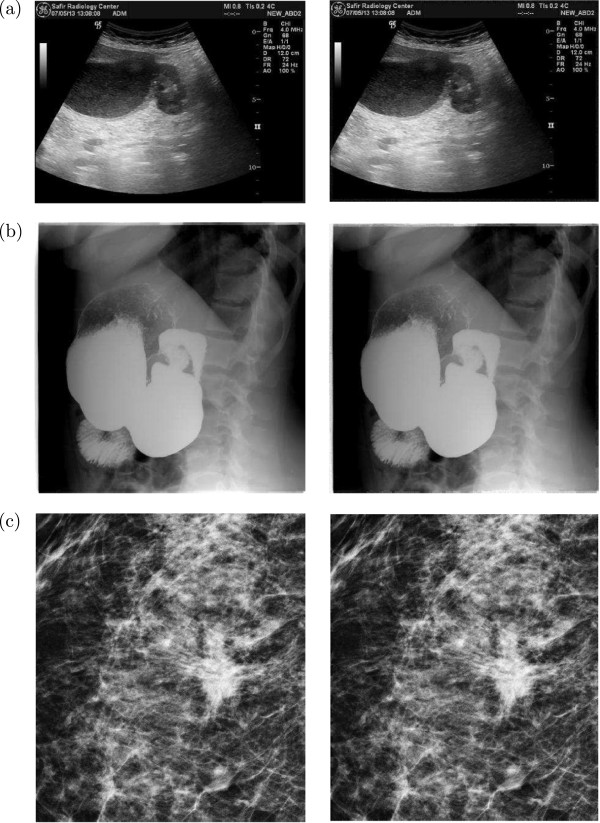
**Original (left column) and watermarked (right column, for** ***N***_***BW***_ ***= 5*** **and** ***N***_***LSB***_ ***= 5*** **) versions of medical images of different modalities: (a) US, (b) RF, and (c) MG**

**Figure 7:**
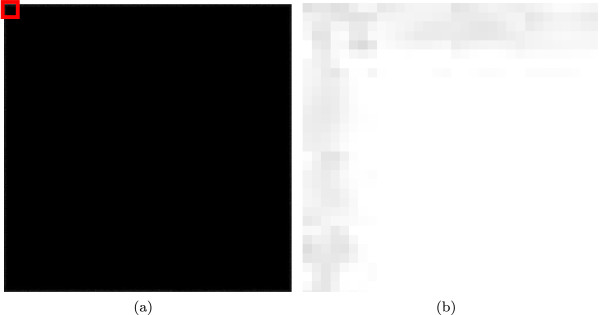
**Absolute difference image of the original and watermarked (** ***N***_***BW***_ ***= 5*** **and** ***N***_***LSB***_ ***= 5*** **) versions of a medical image: (a) full image, and (b) negative of the partial image (as marked at the top-left corner in (a)).**

Examples of the original and watermarked versions of different modality medical images for our embedding scheme are given in Figures 5 and 6. These images not only indicate the imperceptibility of the watermarked images for the proposed embedding scheme, but also support our consideration of border pixels as a general RONI for different modalities of medical images. In order to demonstrate the distortion in the embedding region more clearly, Figure 7 shows a typical case of the absolute difference between the original and watermarked versions of the medical images. This suggests that the embedding region (*i.e.*, RONI) has an almost imperceptible distortion.

### Performance comparison

**Figure 8:**
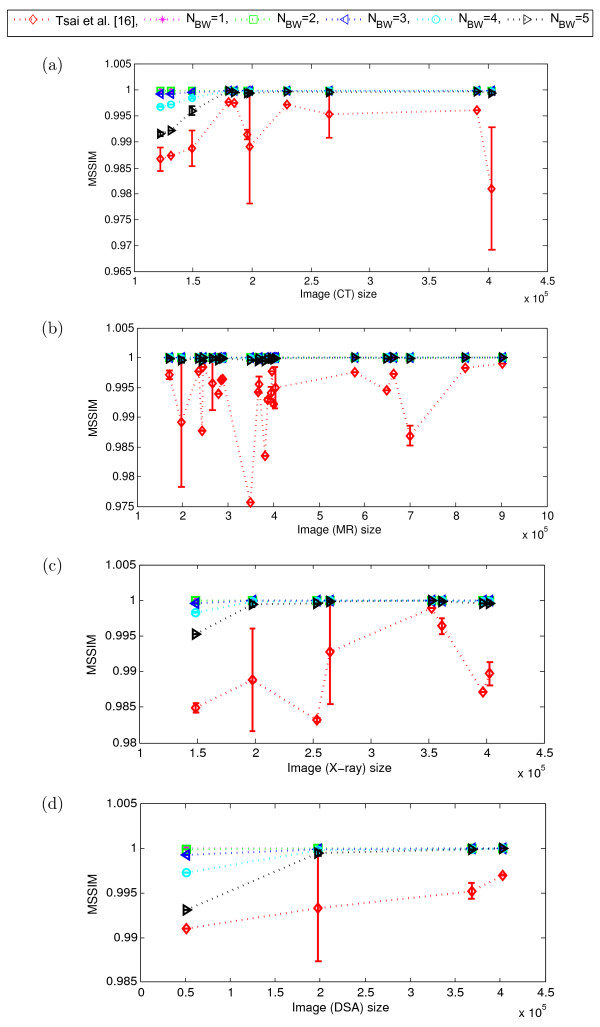
**Perceptual quality (MSSIM) of the proposed scheme and Tsai** ***et al.*** **for different modalities.** **(a)** CT, **(b)** MR, **(c)** X-ray, and **(d)** DSA. (Here, *N*_*LSB*_= 5 and the no. of images in different modalities are: CT = 84, X-ray = 41, MR = 195, DSA = 12. Image sizes are shown in total no. of pixels) [16].

**Figure 9:**
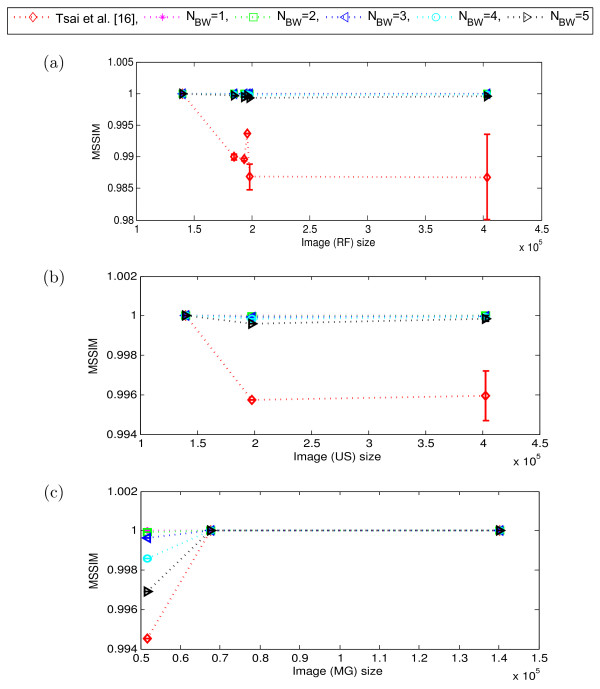
**Perceptual quality (MSSIM) of the proposed scheme and Tsai** ***et al.*** **for different modalities.** **(a)** RF, **(b)** US, and **(c)** MG. (Here, *N*_*LSB*_= 5 and the no. of images in different modalities are: RF = 19, US = 14, and MG = 5. Image sizes are shown in total no. of pixels) [16].

**Figure 10:**
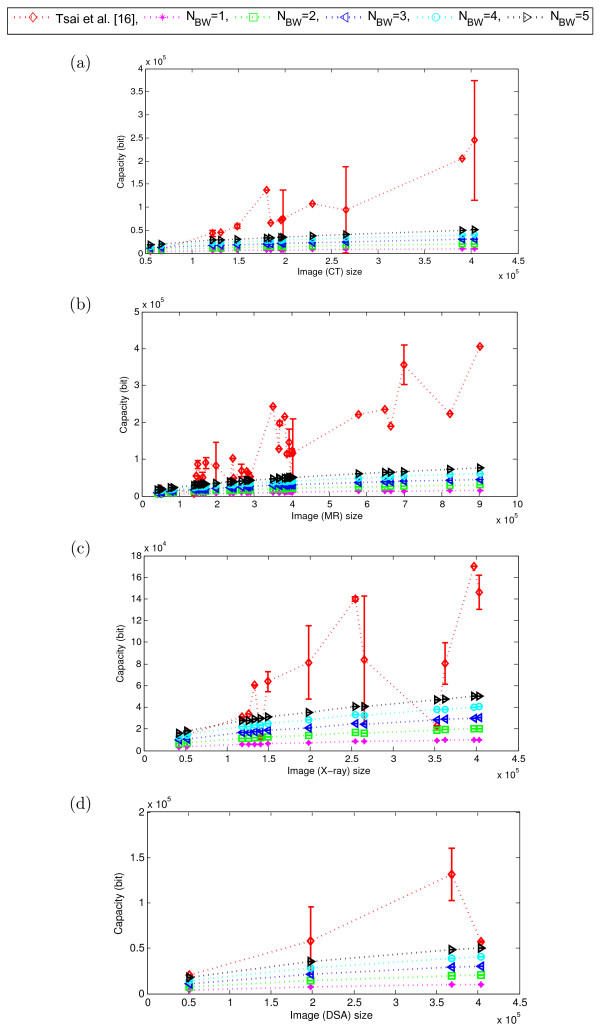
**Embedding capacity (total) of the proposed scheme and Tsai** ***et al.*** **for different modalities.** **(a)** CT, **(b)** MR, **(c)** X-ray, and **(d)** DSA. (Here, *N*_*LSB*_= 5 and the no. of images in different modalities are: CT = 84, X-ray = 41, MR = 195, DSA = 12. Image sizes are shown in total no. of pixels) [16].

**Figure 11:**
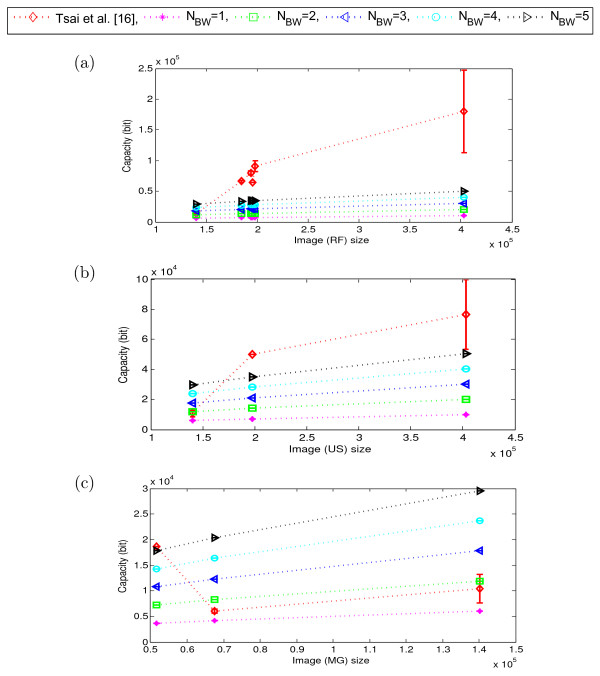
**Embedding capacity (total) of the proposed scheme and Tsai** ***et al.*** **for different modalities.** **(a)** RF, **(b)** US, and **(c)** MG. (Here, *N*_*LSB*_=5 and the no. of images in different modalities are: RF = 19, US = 14, and MG = 5. Image sizes are shown in total no. of pixels) [16].

To validate the potential of content independent embedding over the content dependent embedding for different modalities of medical images, we now compare the performance of our proposed scheme with that of the Tsai *et al.*[16]. As explained in Section “Performance evaluation of the proposed scheme”, we tested our scheme for *N*_*BW*_∈ {1,2,⋯,5}. However, as we observed that up to 5 LSB-planes the embedding impact on quality of the border pixels remains almost imperceptible (Figure 4(d)), we considered here *N*_*LSB*_= 5 and kept it fixed for the rest of our experiments presented in this paper. We examined the variation in performance of the schemes mainly in terms of image quality (MSSIM) and embedding capacity (bits) as illustrated in Figures 8, 9 and Figures 10, 11, respectively. Here, the dotted-lines indicates the trends in MSSIM and embedding capacity variation with the increase of image size, for the Tsai *et al.* scheme and our scheme. The vertical bars in the performance curves indicate the range of variation in the performance (*i.e.*, MSSIM or embedding capacity) of the schemes, for the images of same size (but of different perceptual content). The higher the length of the bar means the wider range of variation in the performance.

The proposed scheme shows a consistent image quality for different modality medical images, which almost linearly varies for the higher image size, which is not the case for the Tsai *et al.* scheme. Figure 8 and Figure 9 show that, for a standard size of 512×512 (which gives 2.6×10^5^ pixels) or higher, our scheme has steadily shown the MSSIM in the range of [ 0.999,1]. Whereas, the MSSIM of Tsai *et al.* scheme randomly varies in a wide range of [ 0.97,1]. In other words, the MSSIM of Tsai *et al.* scheme not only varies with the image size, but it also varies for the same size images. Particularly, for the lower size images (*e.g.*, below the size of 512×512) this randomness and variation are relatively high.

Similarly, the embedding capacity of our scheme linearly increases with the image size, irrespective of the image modalities, whereas that of the Tsai *et al.* scheme does not. Figure 10 and Figure 11 show how the Tsai *et al.* scheme has widely varying embedding capacity, which has different ranges for different modalities. For example, while that scheme has the capacity (bits) range of [50*K*,380*K*] for CT images, the range changes to [20*K*,170*K*] for the X-ray images, as shown in the Figure 10.

Unlike the proposed scheme, this random variation of the MSSIM and embedding capacity of the Tsai *et al.* scheme for different modalities of medical images would not be desirable for the practical applications.

Additionally, the proposed scheme can adaptively control the capacity requirement as discussed in Section “Proposed embedding scheme”, and thus can address the higher embedding capacity requirements. One may argue here that the Tsai *et al.* scheme has higher embedding capacity, which eventually causes more MSSIM drop than our proposed scheme. However, as Table 1 illustrates, generally the MSSIM of the proposed scheme still seems higher than that of the Tsai *et al.* scheme for similar embedding capacity. For example, for 512×512 size images and with *N*_*LSB*_= 5, when Tsai *et al.* scheme has an average capacity of 95.83 Kbits, its MSSIM becomes 0.9895; whereas, the proposed scheme provides the embedding capacity of 100.40 Kbits with MSSIM of 0.9974.

**Table 1:** **Overall Performance comparison for medical images of size** ***512 × 512*** **and** ***N***_***LSB***_ ***= 5***

Methods	MSSIM	Capacity (Kbit)
Tsai *et al.*[16]	0.9895	95.83
Proposed (*N*_*BW*_= 1)	1.0000	10.20
Proposed (*N*_*BW*_= 2)	1.0000	20.40
Proposed (*N*_*BW*_= 3)	0.9999	30.54
Proposed (*N*_*BW*_= 4)	0.9998	40.64
Proposed (*N*_*BW*_= 5)	0.9995	50.70
Proposed (*N*_*BW*_= 6)	0.9992	60.72
Proposed (*N*_*BW*_= 7)	0.9989	70.70
Proposed (*N*_*BW*_= 8)	0.9985	80.64
Proposed (*N*_*BW*_= 9)	0.9980	90.54
Proposed (*N*_*BW*_= 10)	0.9974	100.40

This means that the proposed scheme is more likely to have better capacity-distortion performance than the Tsai *et al.* scheme for higher capacity requirement.

This capacity-distortion performance analysis of our scheme, however, is a separate study and will be addressed in our future research.

The proposed scheme consistently and almost linearly performs over different modalities of medical images. We note here that for a few modalities such as MG, US and DSA, we have used a limited test-set of images. Although the inconsistent performance of Tsai *et al.* scheme is more or less evident in those modalities, our conclusion is based on the other modalities studied in this paper, where we used a relatively high number of test-images.

## Conclusions

In this paper, we proposed a new content-independent embedding scheme for multi-modality medical image watermarking. We generalized RONI, specifying its selection criteria and determining parameters to adaptively control it. While the proposed scheme keeps the ROI pixels in the images untouched, it also maintains a good image quality in the RONI. The proposed scheme avoids the computational overhead of segmentation used for RONI selection in medical images. Our experimental results successfully demonstrated the consistent performance of the proposed scheme for different modalities of medical images. Our findings also suggest that the performance of the state-of-the-art reversible schemes would be modality-wise inconsistent.

As a final remark, with an appropriate watermarking framework (*i.e.*, with the considerations of watermark generation, embedding and detection functions), our proposed scheme can be viable for the multi-modality medical image applications and distant medical services such as teleradiology and eHealth.

In our future work, we will focus on determining suitable limits for the capacity control parameters and the capacity-distortion performance of the proposed scheme to meet higher capacity requirements.

## Endnote

^a^ There are many modalities of medical images. In this paper, we consider the commonly used modalities: Computed Tomography (CT), Magnetic Resonance (MR), X-ray, Digital Subtraction Angiography (DSA), Radio Fluoroscopy (RF), Ultrasound (US), and Mammography (MG).
